# Transcriptomics characteristics and differentiation of subcutaneous adipose tissue among Huainan pigs and its hybrid genetic populations

**DOI:** 10.3389/fvets.2025.1545694

**Published:** 2025-05-22

**Authors:** Taotao Yan, Mingyang Jia, Jiaxi Li, Xianyong Lan, Liwei Yuan, Baosong Xing, Chuanying Pan, Qingxia Lu, Jing Wang

**Affiliations:** ^1^Henan Key Laboratory of Farm Animal Breeding and Nutritional Regulation, Henan Pig Breeding Engineering Research Centre, Institute of Animal Husbandry, Henan Academy of Agricultural Sciences, Zhengzhou, China; ^2^College of Animal Science and Technology, Northwest A&F University, Yangling, Shaanxi, China; ^3^Henan Yifa Animal Husbandry Co, Ltd, Hebi, China

**Keywords:** adipose tissue, Huainan pig, hybridization, transcriptomics, DEGs

## Abstract

**Introduction:**

The Huainan pig (HN) is known for its impressive litter size and exquisite meat quality. However, it also exhibits certain drawbacks such as excessive fat deposition, a relatively low percentage of lean meat percentage, and a slower growth rate. Crossbreeding with lean-type breeds, such as Large White, Landrace, and Berkshire can enhance offspring traits, and increase genetic diversity.

**Methods:**

In this study we employed RNA-seq technology to identify differentially expressed genes (DEGs) in subcutaneous adipose tissue (SAT) samples from HN pigs and their crosses with multiple breeds (with three replicates per group).

**Results:**

In the SAT of Huainan × Berkshire pigs (BH), Huainan × Yorkshire pigs (YH), and Huainan × Landrace pigs (LH), numerous key functional genes were identified, including *LIPG*, *PLIN2*, *CPT1A*, *KLF9*, *CCND1*, *LDLR*, *ACSL1*, *ACLY* and *ANGPTL4*. Functional enrichment analysis revealed that DEGs were primarily involved in several key pathways in BH, including the peroxisome proliferator-activated receptor (PPAR) signaling, metabolic pathways, arachidonic acid metabolism, and arginine/proline metabolism. Similarly, in LH, DEGs were associated with PPAR, cyclic adenosine monophosphate (cAMP) signaling, mitogen-activated protein kinase (MAPK), and the arginine/proline pathway. In contrast, the main pathways in YH were slightly different, including MAPK, fatty acid elongation, arginine/proline metabolism, and glycine/serine/threonine metabolism. Compared to HN, the differential genes in BH, LH, and YH showed a reduced fat deposition. However, in comparison, LH has a stronger subcutaneous fat deposition ability. Notably, LH exhibited a stronger tendency for subcutaneous fat deposition than the other two groups, while YH had the lowest fat deposition capacity.

**Conclusion:**

In conclusion, these findings offer valuable insights and provide a foundation for future research on the molecular mechanisms underlying fat deposition in pigs.

## 1 Introduction

Pork is the most widely produced and consumed meat globally. It serves as a cornerstone of global meat consumption (1). HN pigs (also known Huainan black pigs) are one of the oldest local breeds in northern China. They have all-black bodies, large size, high fertility, strong adaptability, and good meat quality (2). However, this breed exhibits disadvantages such as slow growth, thick back fat, heavy leaf lard, and low lean meat percentage. In contrast, lean-type breeds like Large White, Landrace, and Berkshire pigs grow faster and have higher lean meat yields. These breeds are often used to improve local pig populations (3). For this reason, combining the advantages of these lean breeds with the good traits of HN pigs through crossbreeding, especially to reduce under-skin fat, is critical. Solving this will help improve HN pig breeding and support sustainable pig farming.

Previous research has extensively examined differences in gene activity between lean and fat-type pig breeds. For example, Chen et al. reported that Jiaxing Black pigs (JXB) show significantly higher levels of inositol monophosphate, intramuscular fat, and free amino acids in their longissimus dorsi muscle compared to crossbred pigs (DBJ, Duroc × Berkshire × Jiaxing Black) (4). Additionally, Hang′s revealed distinct differences in the longissimus dorsi muscle between Laiwu pigs (LW, a fat-type breed) and Duroc × Landrace × Yorkshire pigs (DLY, a lean-type breed), particularly in glycerol/phospholipid metabolism. The study also proposed that the PI3K-Akt and MAPK signaling pathways may regulate muscle fiber composition and glucose metabolism (5). Liu demonstrated that Yorkshire pigs (Y) grow faster than Jinhua pigs (J) and their hybrid offspring (YJ), though Yorkshires exhibit lower backfat thickness (6). Similarly, in a comparison of Berkshire pigs, Ningxiang pigs, and their F1 hybrids, researchers identified significant activity in metabolic pathways tied to glycine, serine, threonine, arachidonic acid, fatty acid biosynthesis, linoleic acid, *α*-linolenic acid, and glycerol phospholipid metabolism (7). In conclusion, crossbreeding can effectively enhance local pig breeds by improving key traits such as growth rate, muscle composition, and fat content.

Our previous research has demonstrated that when HN are crossed with Yorkshire, Landrace, and Berkshire pigs, changes occur in the subcutaneous fat deposition of their offspring. However, the molecular mechanisms underlying these differences in subcutaneous fat deposition between HN pigs and their crosses with these breeds remain poorly understood. Therefore, this study utilizes transcriptome sequencing technology to investigate the regulatory mechanisms influencing subcutaneous fat deposition in the offspring resulting from the crossbreeding of HN pigs with Yorkshire, Landrace, and Berkshire pigs, and to identify key genes that may affect subcutaneous fat thickness. This study provides valuable insights into the genetic mechanisms underlying fat deposition in pigs, offering guidance for improving selection strategies for backfat thickness and adipose tissue traits in pig breeding programs.

## 2 Materials and methods

### 2.1 Ethical statement

The experimental animals used and the operations performed in this study were approved by the Institute of Animal Science, Henan Academy of Agricultural Sciences (code May 2, 2015), in accordance with its guidelines (8).

### 2.2 Materials and sample collection

The hybridization experiment was conducted at the Huainan Original Breeding Farm of Henan Xingrui Agriculture and Animal Husbandry Technology Co., Ltd. In this experiment, we selected 60 Huainan sows of similar age and body condition and randomly divided them into three groups, with 20 sows in each group. These sows were bred using semen from boars of the Yorkshire, Landrace, and Large White breeds, creating three hybrid combinations: Yorkshire-Huainan (YH), Landrace-Huainan (LH), and Large White-Huainan (BH). From these three hybrid offspring groups, as well as the purebred group (HN), we selected 10 castrated male pigs, each weighing approximately 30 kg, for a fattening trial. These selected pigs were of similar age and body condition. When these pigs reached the market-standard weight of approximately 100 kilograms, three pigs were randomly selected from each group for standardized slaughter. After measuring their carcass traits, samples of subcutaneous adipose tissue were collected and rapidly frozen in liquid nitrogen for subsequent experimental analysis.

### 2.3 RNA extraction, library construction, and transcriptome sequencing

Total RNA was extracted using TRIzol^™^ Reagent (Thermo Fisher Scientific, Cat#15596018) and assessed for quantity/purity with a Bioanalyzer 2100 system and RNA 6000 Nano LabChip Kit (Agilent Technologies). High-quality RNA samples with RNA Integrity Number (RIN) ≥ 7.0 were selected for library preparation. mRNA was isolated from 5 μg of total RNA through two rounds of purification with Dynabeads^™^ Oligo (dT) beads. Fragmentation of mRNA was performed via divalent cation-mediated cleavage at elevated temperatures, followed by reverse transcription into cDNA. Unlabeled second-strand DNA was synthesized, and adenine (A) overhangs were added to blunt ends to enable ligation with indexed adapters. Dual-indexed adapters (Illumina) were ligated, and size selection was conducted using AMPure XP beads. After uracil-DNA glycosylase treatment, PCR amplification was performed under optimized cycling conditions. Final cDNA libraries exhibited an average insert size of 300 ± 50 bp and were sequenced in 2 × 150 bp paired-end mode on an Illumina NovaSeq^™^ 6,000 platform.

### 2.4 Raw data processing, transcriptome assembly

The raw data were initially processed through quality filtering using Cutadapt (v1.9)^1^ to remove adapter sequences and low-quality reads. Quality assessment was subsequently performed using FastQC, generating clean reads for downstream analysis. These quality-controlled reads were then aligned to the porcine reference genome using HISAT2 (v2.2.1).^2^ Transcript assembly was conducted *de novo* using StringTie (v2.1.6),^3^ followed by integration of assembly results across all samples. The assembled transcripts were systematically annotated and classified using gffcompare (v0.9.8).^4^

### 2.5 DEGs analysis

In this study, we first identified differentially expressed genes (DEGs) between the two sample groups using DESeq2, followed by significance validation of these DEGs through edgeR analysis. The genes with the parameter of *p*-value below 0.05 and absolute fold change ≥ 2 were considered differentially expressed genes.

### 2.6 Gene Ontology and Kyoto Encyclopedia of Genes and Genomes pathway enrichment analysis

The functional annotation of differential genes was conducted using the Gene Ontology (GO) database.^5^ The number of differential genes included in each GO term was counted, and the significance of their enrichment was calculated using the hypergeometric distribution algorithm. The stem ranking method was utilized to select the most significantly enriched functional terms (9). Additionally, pathway analysis of differential protein-coding genes was performed using the Kyoto Encyclopedia of Genes and Genomes (KEGG) database (10).^6^ The *p*-value was calculated to assess the significance of enrichment, with pathways having a *p*-value of less than 0.05 considered to be significantly enriched in DEGs.

### 2.7 Correlation analysis of replicas

We used R software to perform correlation analysis. The Pearson correlation coefficient between two replicas was calculated to evaluate repeatability between samples. The closer the correlation coefficient gets to 1, the better the repeatability between two parallel experiments.

### 2.8 Gene set enrichment analysis

Using gene set enrichment analysis (GSEA) (version 4.1.0) and MSigDB, we conducted gene set enrichment analysis to compare gene expression between two groups. Our analysis focused on GO terms, KEGG pathways, DO terms, and Reactome pathways. Genes were ranked using Signal2Noise normalization, and enrichment scores and *p*-values were calculated with the default parameters. GO terms, KEGG pathways, DO terms, and Reactome pathways with |NES| > 1, NOM *p*-value < 0.05, and FDR *q*-value < 0.25 were considered significantly different between the groups.

### 2.9 Protein–protein interaction

To clarify the interaction relationships among the differentially expressed genes (DEGs) encoding proteins, we selected DEGs identified in three pairwise comparisons (HN vs. BH, HN vs. LH, and HN vs. YH) with an adjusted p-value <0.05 and |log2 fold change| > 1. We utilized the STRING database^7^ to analyze the associations between the proteins encoded by these DEGs, setting the minimum required interaction score to 0.5 and hiding disconnected nodes in the network. The protein–protein interaction (PPI) data generated by STRING was then imported into Cytoscape (v3.9.1) for further visualization of the PPI network among these DEGs.

### 2.10 Quantitative reverse transcription polymerase chain reaction

In this study, after RNA-seq analysis of the subcutaneous adipose tissue from Huainan pigs and their crosses, we selected genes *DPP6*, *DNAJB6*, *HOXC10*, *SLC7A10*, *CD163*, *LDLR*, *CPT1A*, and *WNT9B* for validating the RNA-seq results. These genes exhibited extremely significant differences in BH, LH, and YH compared to HN and were enriched in signaling pathways related to fat deposition. Total RNA was extracted from porcine subcutaneous adipose tissue with AG RNAex Pro RNA extraction reagent. After determining the nucleic acid concentration, RNA was reverse transcribed using Hifair^®^ III 1st Strand cDNA Synthesis SuperMix for qPCR (gDNA digester plus). Next, quantitative reverse transcription polymerase chain reaction (qRT-PCR) analysis was carried out with ChamQ SYBR qPCR Premix (Nanjing Vazyme Biotech Co., Ltd.). The 13 μL reaction mixture contained 6.5 μL of 2 × Premix, 0.5 μL each of forward and reverse primers, 5 μL of cDNA diluted 10-fold, and 0.5 μL of ddH_2_O. The thermal cycling conditions were: 95°C for 30 s (initial denaturation), followed by 40 cycles of 95°C for 10 s (denaturation) and 60°C for 30 s (annealing). A melting curve analysis was done after cycling to confirm amplification specificity. Relative gene expression levels were calculated via the 2^−ΔΔCt^ method. The sample size for subcutaneous adipose tissue of HN, BH, LH, and YH was three per group. Tissue samples used in the experiment and sequencing came from the same individuals. Three technical replicates were used, with Glyceraldehyde-3-Phosphate Dehydrogenase (*GAPDH*) as the housekeeping gene. The primer sequences are listed in Table 1.

**Table 1:** Primers for real-time fluorescent quantitative PCR.

Gene accession number	Genes	Primers (5′-3′)	Product length
XM_003127014.5	SLC7A10	F: GCCAGGAGCTGTGTTTCGT	132 bp
		R: TTCAGGCTCCACAAGTGTCTC	
NM_001244430.1	DNAJB1	F: GAACCCCGATGGAAAGAGCA	132 bp
		R: TCGGCTGGAATGTTGTTGGA	
NM_001129805.1	CPT1A	F: GCCTCTACGTGGTGTCCAAA	79 bp
		R: GATAATCGCCACGGCTCAGA	
XM_021091726.1	HOXC10	F: TGACATGCCCTCGCAATGTA	89 bp
		R: AGCTGCGCTCCGGCTAT	
XM_063238127.1	CD163	F: TTGGCCTGTCTCATCGCATT	173 bp
		R: TCAGAGTGGTCTCCTGAGGG	
XM_021066827.1	WNT9B	F: CCCAGCCTTGGTGAGGATTT	81 bp
		R: AGTCCAGCCCTCCTCTCAAT	
XM_021079151.1	DPP6	F: GTTCTTCTTCGTCCGAGCCA	135 bp
		R: ATTGTGGTCACGTCCCAGTC	
NM_001206354.2	LDLR	F: AAGAGCGCACACAGTGAACA	163 bp
		R: CGGCACTGGAACTCGTTTCT	
NM_001206359.1	GAPDH	F: CCCCGTTCGACAGACAGC	94 bp
		R:GATGCGGCCAAATCCGTTCAC	

### 2.11 Statistical analysis

The comparison between two groups was conducted using an unpaired two-tailed Student’s t-test, while comparisons among multiple groups were performed using one-way ANOVA. *p* < 0.05 was considered statistically significant for differences. The statistical analysis was conducted using GraphPad Prism 8.0 software.

## 3 Results

### 3.1 A comparison of carcass traits between Huainan pigs and their hybrids

**Figure 1:**
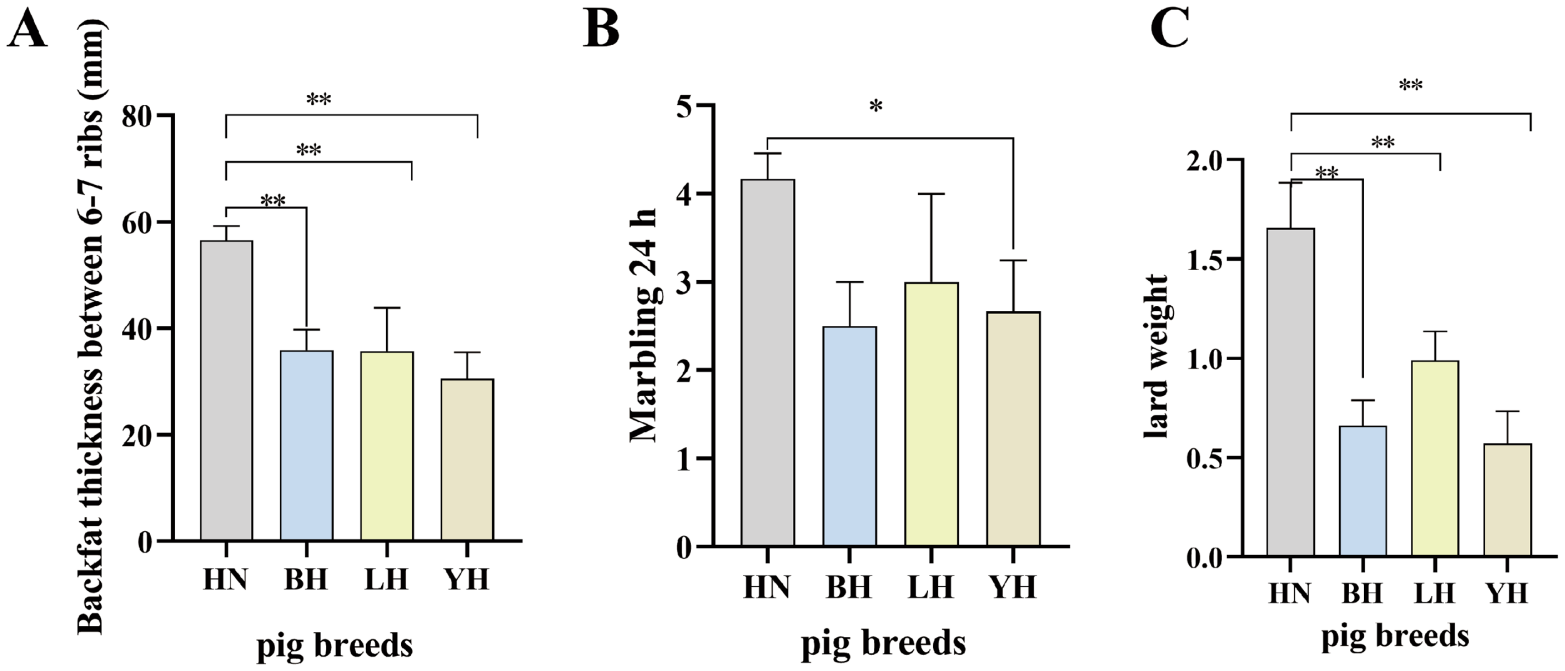
Comparative analysis of HN and three crosses (BH, LH, YH) in terms of: **(A)** backfat thickness (mm) measured between the 6th and 7th ribs, **(B)** marbling score after 24 h, and **(C)** leaf lard weight. “*” denotes a statistical significance of *p* < 0.05. “**” denotes a statistical significance of *p* < 0.05.

All pigs were reared under identical conditions with free access to feed and water. This study compared the backfat thickness between the 6th and 7th ribs, 24-h marbling score, and leaf lard weight among HN and three crosses (BH, LH, YH). The results showed that, compared to HN, the backfat thickness between the 6th and 7th ribs of the three crosses (BH, LH, YH) was significantly reduced (*p* < 0.01) (Figure 1A), as well as their leaf lard weight (*p* < 0.01) (Figure 1B). Additionally, the 24-h marbling score of BH was significantly lower than that of HN (*p* < 0.05) (Figure 1C).

### 3.2 Overview of sequencing data

**Figure 2:**
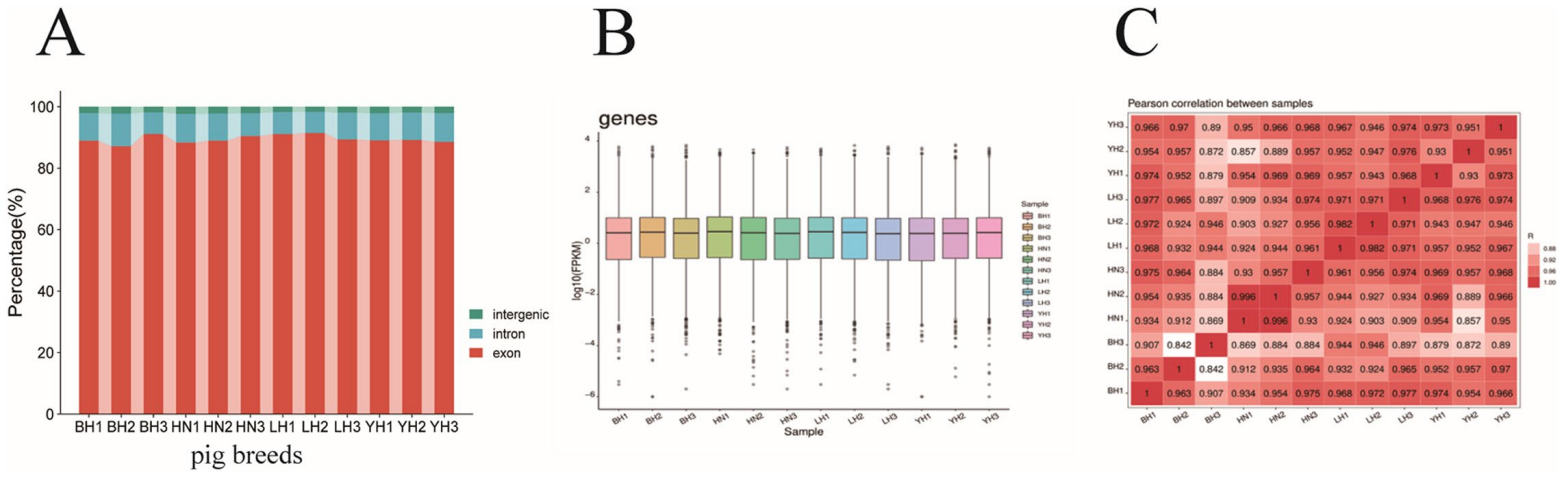
Comparison of sequencing reads and distribution of positively expressed genes. **(A)** The distribution of sequencing reads in the reference genome. The distribution of reads in the exons (red), introns (blue), and intergenic regions (green) are shown. **(B)** Distributions of expression values of 12 samples. The boxplots show log10 (FPKM + 1) values of each gene from the 4 sets of RNA-Seq data. The black lines in the boxes represent the medians. **(C)** Pearson correlation coefficients for comparisons among all samples.

After sequencing 12 subcutaneous adipose tissue samples, the raw read data obtained ranged from 34527940 to 45232150 kb, with original sequence lengths all exceeding 5.18 gigabases (G). After quality control and filtering, the clean read data ranged from 33082598 to 43586246, with sequence lengths for each sample exceeding 4.96 G. The percentages of Q20 and Q30 bases exceeded 99.72% and 97.16%, respectively, and the average GC content for all samples was 50.00% (Table 2). When comparing the transcriptome data with the reference genome, we found that the aligned read data accounted for more than 94% (ranging from 94.79% to 96.57%). Among these, an average of 89.49% of the read data aligned to exon regions, 8.51% aligned to intron regions, and 2.0% aligned to intergenic regions (Figure 2A). The expression distributions of protein-coding genes were similar across all samples (Figure 2B). In this study, the R^2^ values between biological replicates within each group were all greater than 0.84 (Figure 2C), indicating that the data is reliable and suitable for further analysis.

**Table 2:** Sample quality control statistics data.

Sample	Raw reads (kb)	Raw base (G)	Clean reads (kb)	Clean base (G)	Mapped reads (%)	Q20 (%)	Q30 (%)	GC content (%)
HN1	34527940	5.18	33082598	4.96	95.81	99.73	97.18	49.50
HN2	43297806	6.49	41252724	6.19	95.28	99.72	97.16	49.50
HN3	40457854	6.07	38349308	5.75	94.79	99.74	97.21	49.50
BH1	34861420	5.23	33487360	5.02	96.06	99.78	98.37	50.00
BH2	35148098	5.27	33841794	5.08	96.28	99.76	98.40	49.00
BH3	39384714	5.91	38016996	5.70	96.53	99.78	98.33	50.50
LH1	38395664	5.76	37023718	5.55	96.43	99.78	98.37	50.50
LH2	39629948	5.94	38271138	5.74	96.57	99.78	98.26	50.50
LH3	45232150	6.78	43586246	6.54	96.36	99.79	98.50	49.50
YH1	42524214	6.38	40624370	6.09	95.53	99.76	98.19	50.50
YH2	36394292	5.46	35147434	5.27	96.57	99.78	98.45	49.50
YH3	37746248	5.66	36426646	5.46	96.50	99.79	98.44	49.50

### 3.3 Identification of DEGs

**Figure 3:**
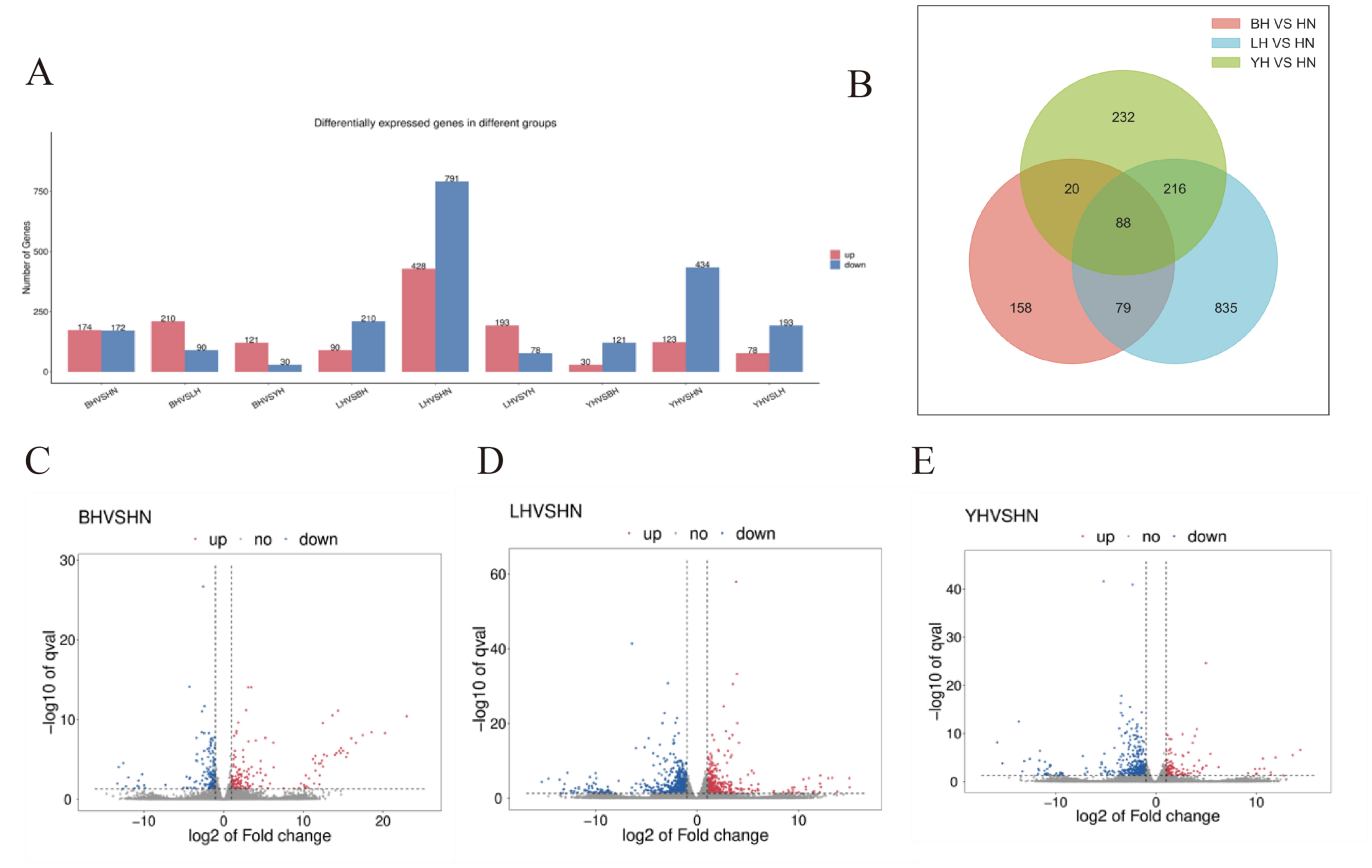
Differential gene expression analysis of subcutaneous adipose tissue between different groups. **(A)** Bar plot of differentially expressed genes in different groups. **(B)** Venn diagram of DEGs in different groups. **(C–E)** Volcano plots of Volcano map of DEGs.

To analyze the differences in gene expression in subcutaneous adipose tissue between the HN pigs and three crosses (BH, LH, YH) we conducted a statistical analysis of DEGs based on transcriptome data. Compared to the HN group, 346, 1,219, and 557 DEGs were identified in the BH, LH, and YH groups, respectively. Among them, the BH group had 174 upregulated genes and 172 downregulated genes; the LH group had 791 downregulated genes and 428 upregulated genes; and the YH group had 434 downregulated genes and 123 upregulated genes. According to the Venn diagram, there were 167 common DEGs between the BH and LH groups, 108 common DEGs between the BH and YH groups, and 304 common DEGs between the LH and YH groups. Additionally, there were 88 common DEGs shared among all three groups of BH, LH, and YH (Figures 3A,B). Subsequently, volcano plots were constructed to visually display the distribution of these significantly differentially expressed genes (Figures 3C–E). The results indicated that when HN was crossed with Berkshire, Landrace, and Yorkshire pigs, respectively, the majority of DEGs in the LH and YH groups were downregulated, with the LH group having the highest number of DEGs. In contrast, the number of upregulated and downregulated genes in the BH group was nearly equal.

### 3.4 Differential gene screening and identification

**Figure 4:**
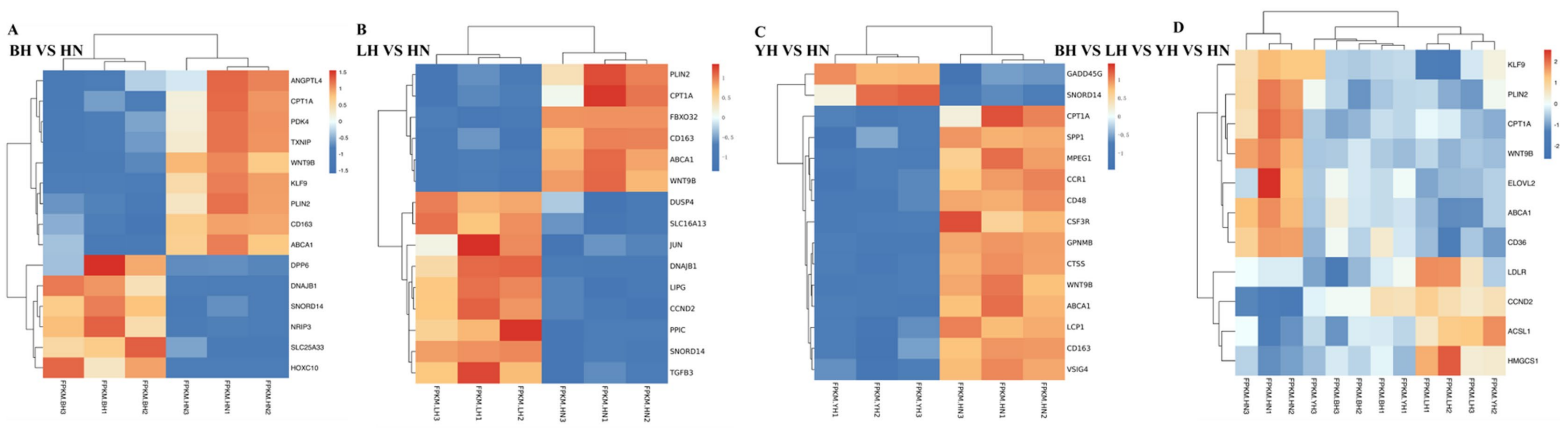
Heatmap of differentially expressed genes (DEGs) in subcutaneous adipose tissue across porcine hybrid groups. **(A)** BH vs. HN; **(B)** LH vs. HN; **(C)** YH vs. HN; **(D)** BH vs. LH vs. YH vs. HN.

To identify genes specifically regulating subcutaneous fat deposition, we compared the DEGs between HN pigs and BH, LH, and YH pigs. Our results indicate that, compared to the HN group, the BH, LH, and YH groups exhibited downregulated expression of peroxisome *PPAR* target genes, including Carnitine palmitoyl transferase 1A (*CPT1A*), Platelet glycoprotein 4 (*CD36*), Angiopoietin-like 4 (*ANGPTL4*), and Kruppel-like factor 9 (*KLF9*); lipid metabolism genes such as ATP-Binding Cassette Transporter A1 (*ABCA1*); and genes related to cell differentiation and development, namely Wnt family member 9B (*WNT9B*) and Perilipin 2 (*PLIN2*). Conversely, these groups showed upregulated expression of fatty acid synthesis and metabolism-related genes, including Low-density lipoprotein receptor (*LDLR*), Acyl-CoA synthetase long-chain family member 1 (*ACSL1*), and 3-Hydroxy-3-methylglutaryl-CoA synthase 1 (*HMGCS1*) (Figure 4D). Additionally, compared to the HN group, the BH group exhibited downregulated expression of metabolism regulation-related genes such as Pyruvate dehydrogenase kinase isoenzyme 4 (*PDK4*) and Thioredoxin-interacting protein (*TXNIP*), whereas *TXNIP* and Homeobox C10 (*HOXC10*) were upregulated. In the LH group, upregulated expression was observed for cell cycle-related proteins such as *Cyclin D2* (*CCND2*) and *Cyclin D1* (*CCND1*); lipid metabolism genes including Lipoprotein lipase (*LIPG*) and Solute carrier family 16 member 13 (*SLC16A13*); and transcription factors such as *JUN*, DnaJ heat shock protein subfamily B member 1 (*DNAJB1*), and Dual specificity phosphatase 4 (*DUSP4*). Meanwhile, in the YH group, downregulated expression was noted for macrophage markers such as CD163 Molecule (*CD163*) and Macrophage expressed gene 1 (*MPEG1*); chemokine receptors including C-C chemokine receptor type 1 (*CCR1*); CSF3 receptor (*CSF3R*); and Lymphocyte cytosolic protein 1 (*LCP1*) (Figures 4A–C). In summary, the aforementioned differentially expressed genes may regulate subcutaneous adipose tissue deposition in the HN, BH, LH, and YH groups through various biological pathways.

### 3.5 Functional analysis of DEGs

**Figure 5:**
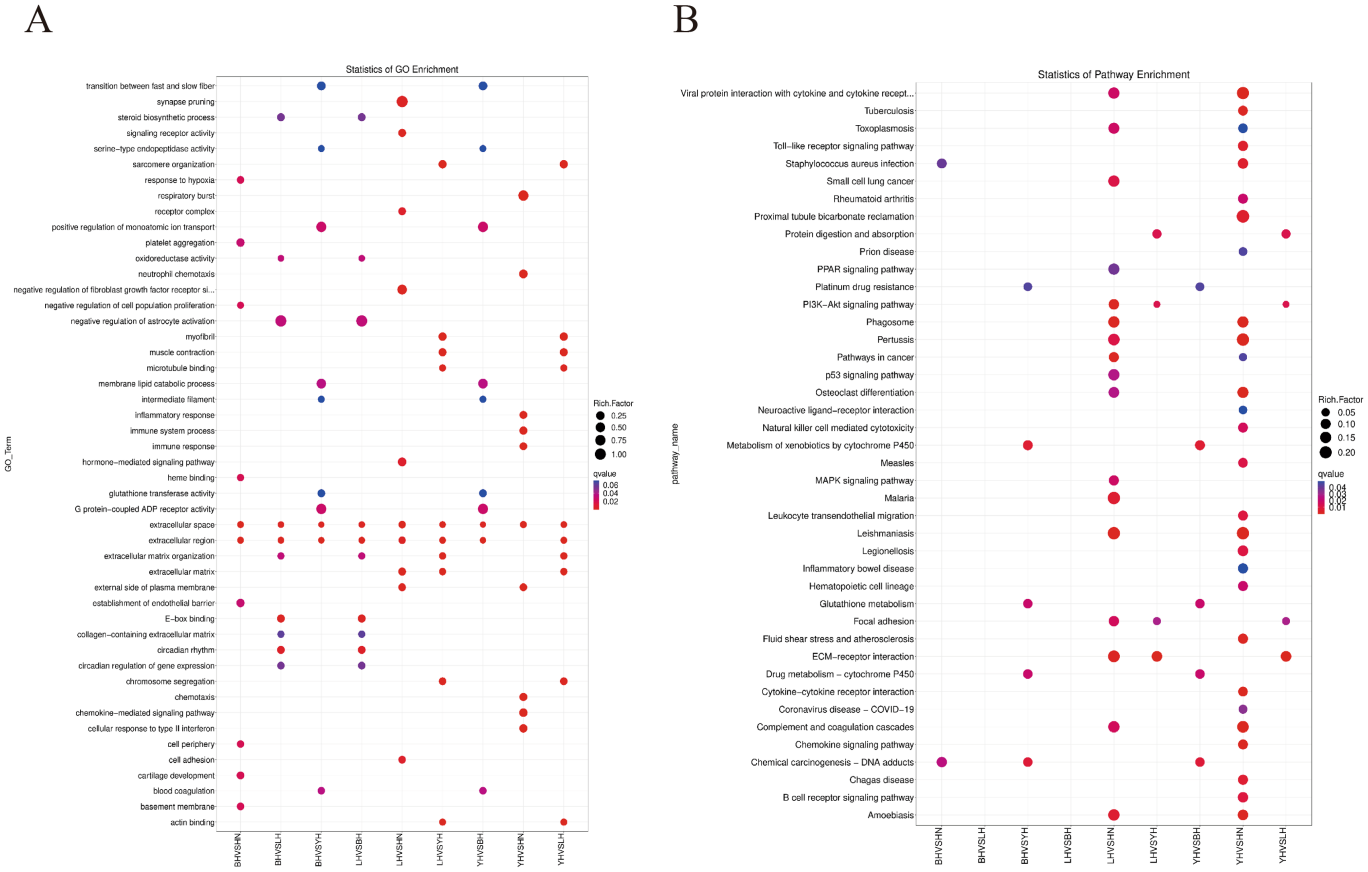
Functional enrichment analysis of differentially expressed genes. **(A)** Gene Ontology (GO) term enrichment; **(B)** Kyoto Encyclopedia of Genes and Genomes (KEGG) pathway enrichment.

**Figure 6:**
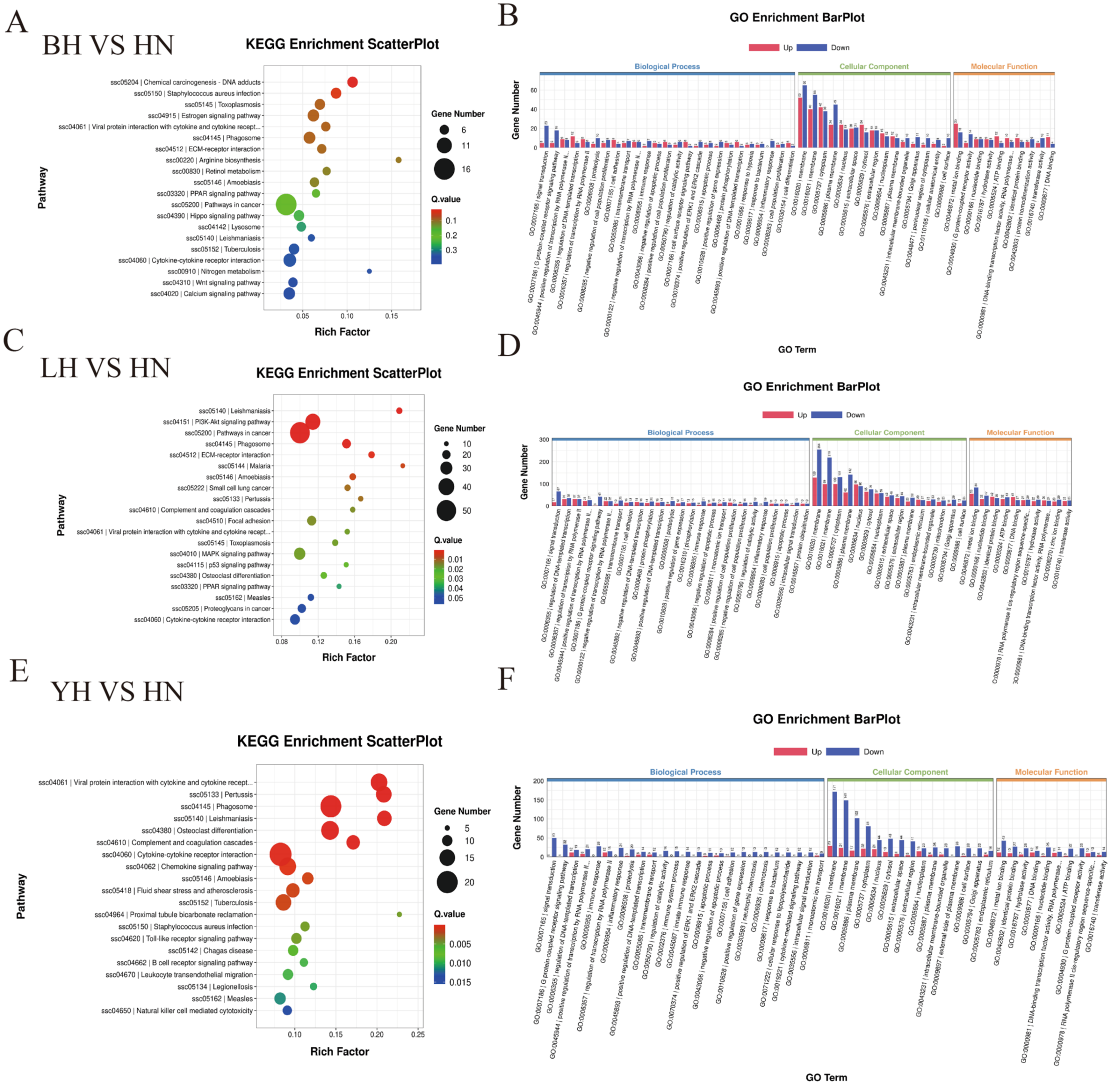
GO database enrichment analysis of DEGs: **(A,B)** LH vs. HN, **(C–D)** BH vs. HN, **(E,F)** YH vs. HN.

**Figure 7:**
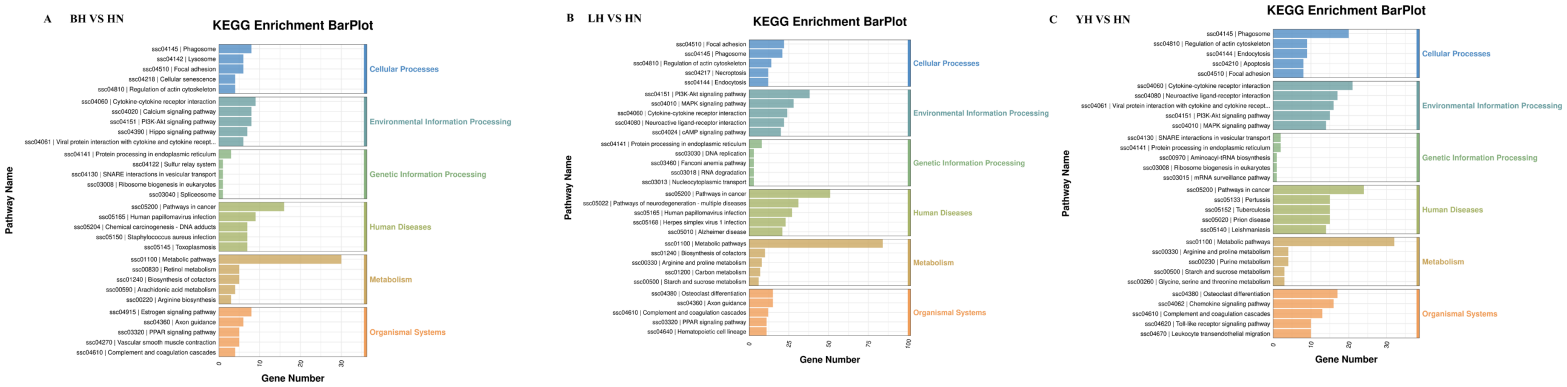
KEGG enrichment analysis of DEGs. **(A)** BH vs. HN; **(B)** LH vs. HN; **(C)** YH vs. HN.

To further investigate the functional characteristics of DEGs between the HN and three crosses (BH, LH, and YH), we annotated the identified genes using the GO and KEGG databases (Figure 5). The results of GO enrichment analysis showed that these DEGs were categorized into three major groups: biological processes, cellular components, and molecular functions. The GO enrichment analysis revealed that the DEGs between the HN and the three crosses (LH, BH, YH) were significantly enriched in biological processes such as the extracellular region and extracellular matrix organization, hormone-mediated signaling pathways, cell adhesion, external side of plasma membrane, synaptic pruning, serine-type endopeptidase activity, and glutathione transferase activity (Figure 6). The KEGG results indicated that, compared to the HN group, a total of 135 DEGs in the BH group were association with 224 pathways, 425 DEGs in the LH group were association with 300 pathways, and 214 DEGs in the YH group were annotated into 257 pathways. Among the top 20 metabolic pathways with significant enrichment (*p* ≤ 0.05), the pathways enriched by DEGs in the BH group included calcium signaling pathway, Hippo signaling pathway, PPAR signaling pathway, metabolic pathways, arachidonic acid metabolism, and arginine and proline metabolism (Figure 7A). In the LH group, significantly enriched pathways included cAMP signaling pathway, MAPK signaling pathway, PPAR signaling pathway, arginine and proline metabolism, and carbon metabolism (Figure 7B). The pathways enriched by DEGs in the YH group included MAPK signaling pathway, metabolic pathways, arginine and proline metabolism, and glycine, serine, and threonine metabolism (Figure 7C). In summary, after crossing HN with Berkshire, Landrace, and Yorkshire pigs, the DEGs were significantly enriched in key signaling pathways such as PI3K-Akt, MAPK, and PPAR.

### 3.6 GESA enrichment analysis

**Figure 8:**
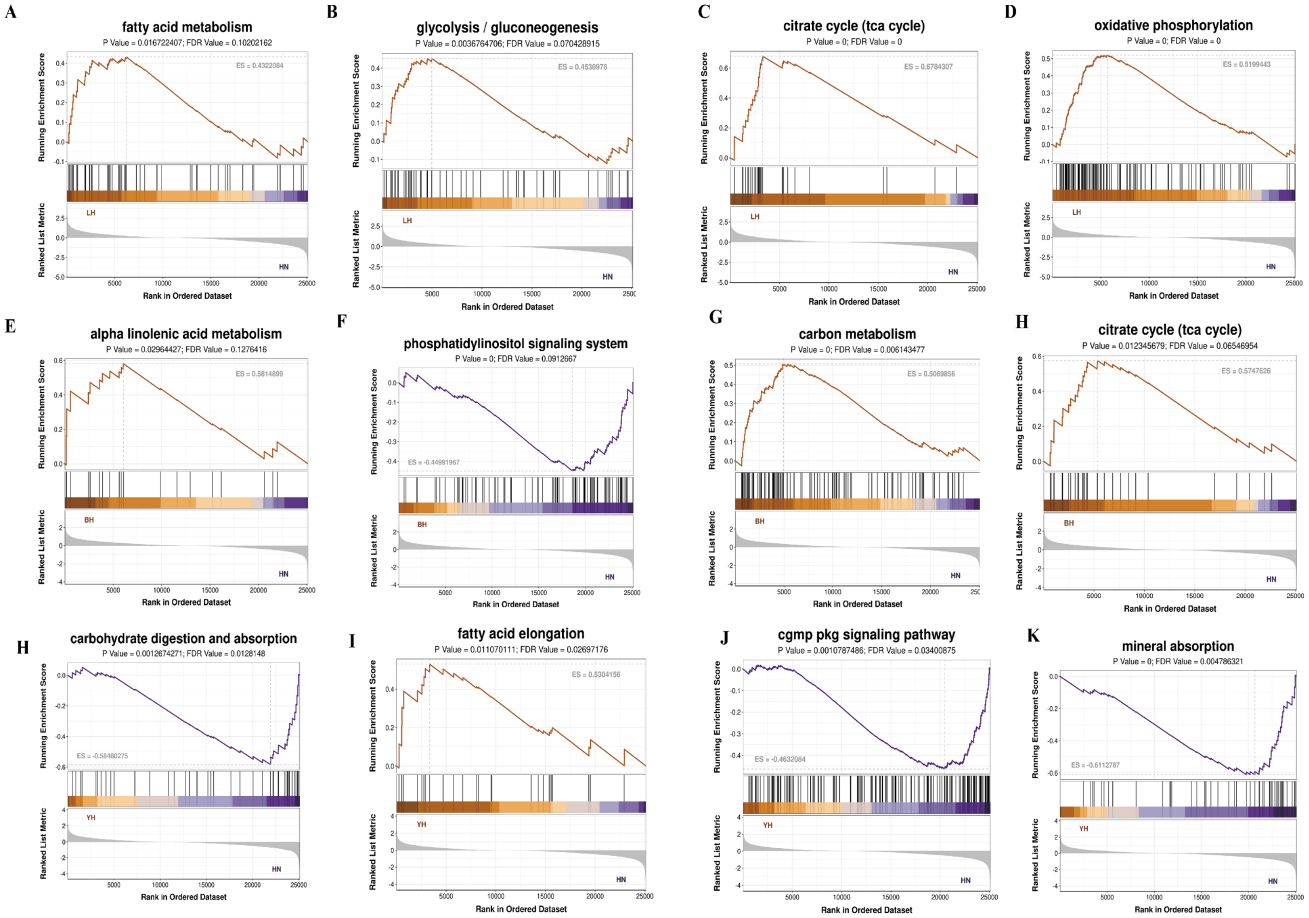
GESA enrichment analysis of all the DEGs. **(A–D)** BH vs. HN; **(E–H)** LH vs. HN; **(I–L)** YH vs. HN.

Next, we performed GSEA to investigate the enrichment patterns of DEGs across HN pigs and three crosses (BH, LH, and YH) within key biological pathways and disease-related processes. These pathways included lipid metabolism (such as arachidonic acid and linoleic acid metabolism), amino acid metabolism (glycine, serine, threonine, and glutamate metabolism), energy metabolism (citric acid cycle, oxidative phosphorylation, carbon metabolism), biosynthesis (steroid and aminoacyl-tRNA biosynthesis), immune regulation (NF-κB and PI3K-Akt signaling pathways), and cellular signal transduction (MAPK signaling pathway) (Figure 8). When comparing BH with HN, genes linked to *α*-linolenic acid metabolism, carbon metabolism, as well as glycine, serine, and threonine metabolism, and arachidonic acid metabolism, all demonstrate a general upregulation trend (Figures 8A–D). Similarly, in the comparison of LH to HN, genes involved in fatty acid metabolism, steroid biosynthesis, the tricarboxylic acid cycle (TCA cycle), and carbon metabolism exhibit an upregulation trend (Figures 8E–H). In contrast, YH vs. HN comparisons exhibited a distinct pattern: genes linked to carbohydrate digestion/absorption and the NF-κB signaling pathway were downregulated, while those involved in fatty acid elongation and carbon metabolism were upregulated (Figures 8I–K).

### 3.7 Analysis of the network of DEGs

**Figure 9:**
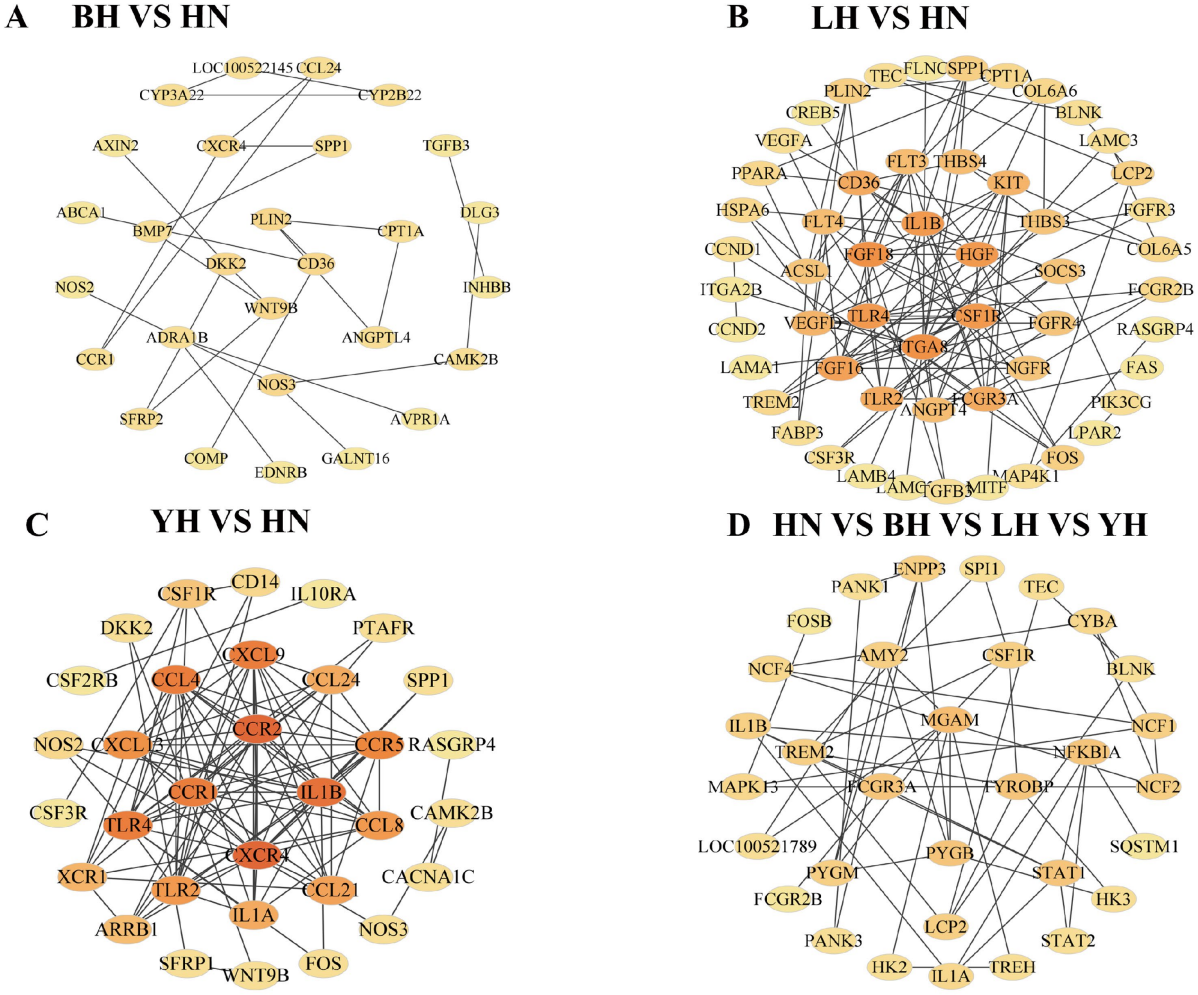
Differential expression gene interaction network. **(A)** BH vs. HN; **(B)** LH vs. HN; **(C)** YH vs. HN; **(D)** The common DEGs shared among the three groups: BH vs. HN, LH vs. HN, and YH vs. HN.

To further identify the key genes associated with subcutaneous fat deposition in HN pigs and the three crosses (BH, LH, and YH), we constructed a PPI network using Cytoscape based on the target gene list extracted from the STRING database (Figure 9). Compared to HN, specifically, in BH (*CD36*, WTN9B, *PLIN2*, *DKK2*), LH (*FGF18*, *CSF1R*, *HGF*, *IL1B*), and YH (*CCR1*, *CCR2*, *CXCR1*, *IL1B*), the following genes are identified as core hub genes. Among the common DEGs shared by the three groups (BH vs. HN, LH vs. HN, YH vs. HN), *FCGR3A*, *PYGB*, *MGAM*, and *TYROBP* are identified as core hub genes. Additionally, we observe several significant interactions between *PLIN2* and *CD36*/*WNT9B*, as well as between *CPT1A* and PLIN2.

### 3.8 Validation of eight differentially expressed genes: *DPP6, DNAJB1, HOXC10, SLC7A10, CD163, LDLR, CPT1A,* and *WNT9B*

**Figure 10:**
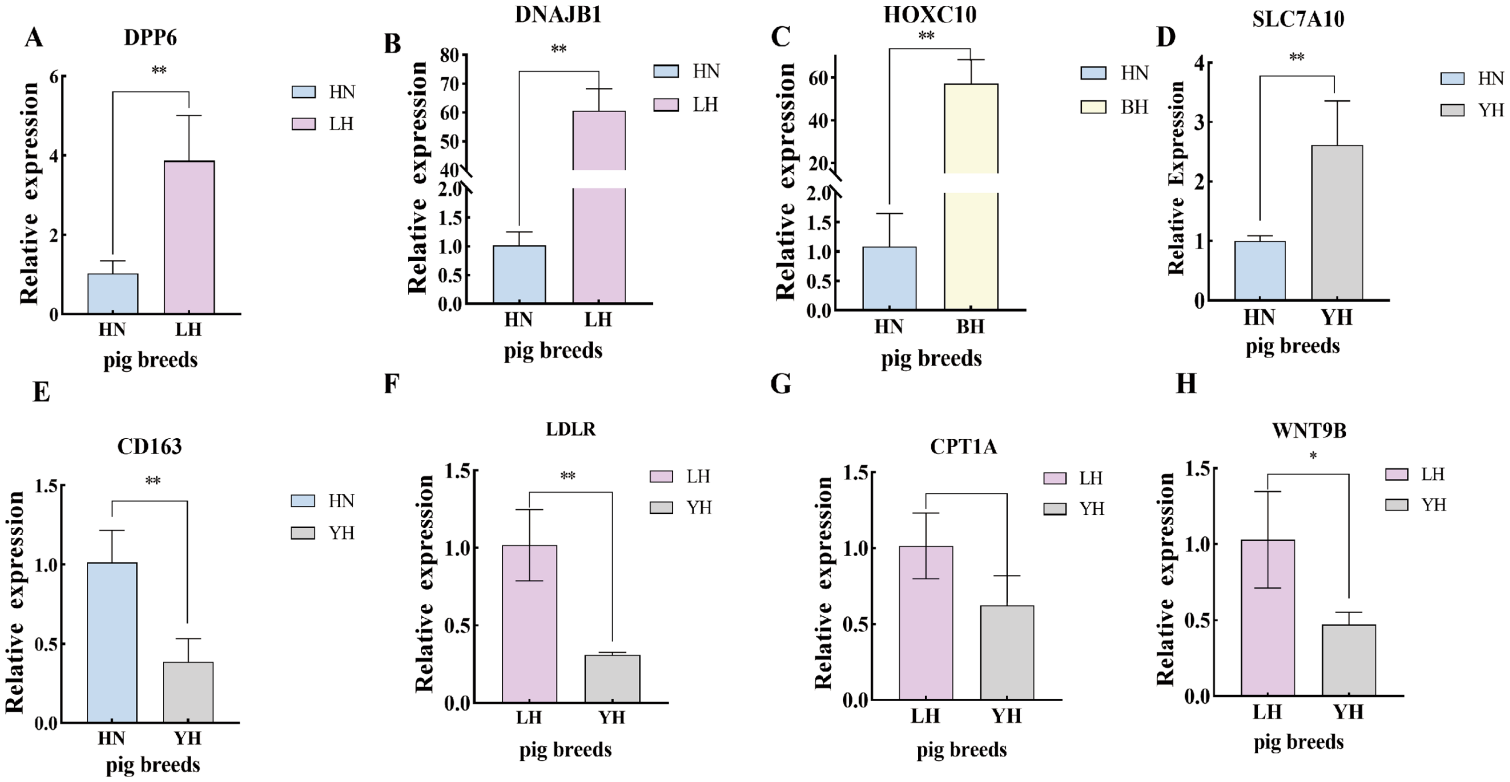
Validation of the DEGs using qRT-PCR. **(A–H)** qRT-PCR verification of differential gene expression. ^*^p < 0.05, ^**^p < 0.01.

**Figure 11:**
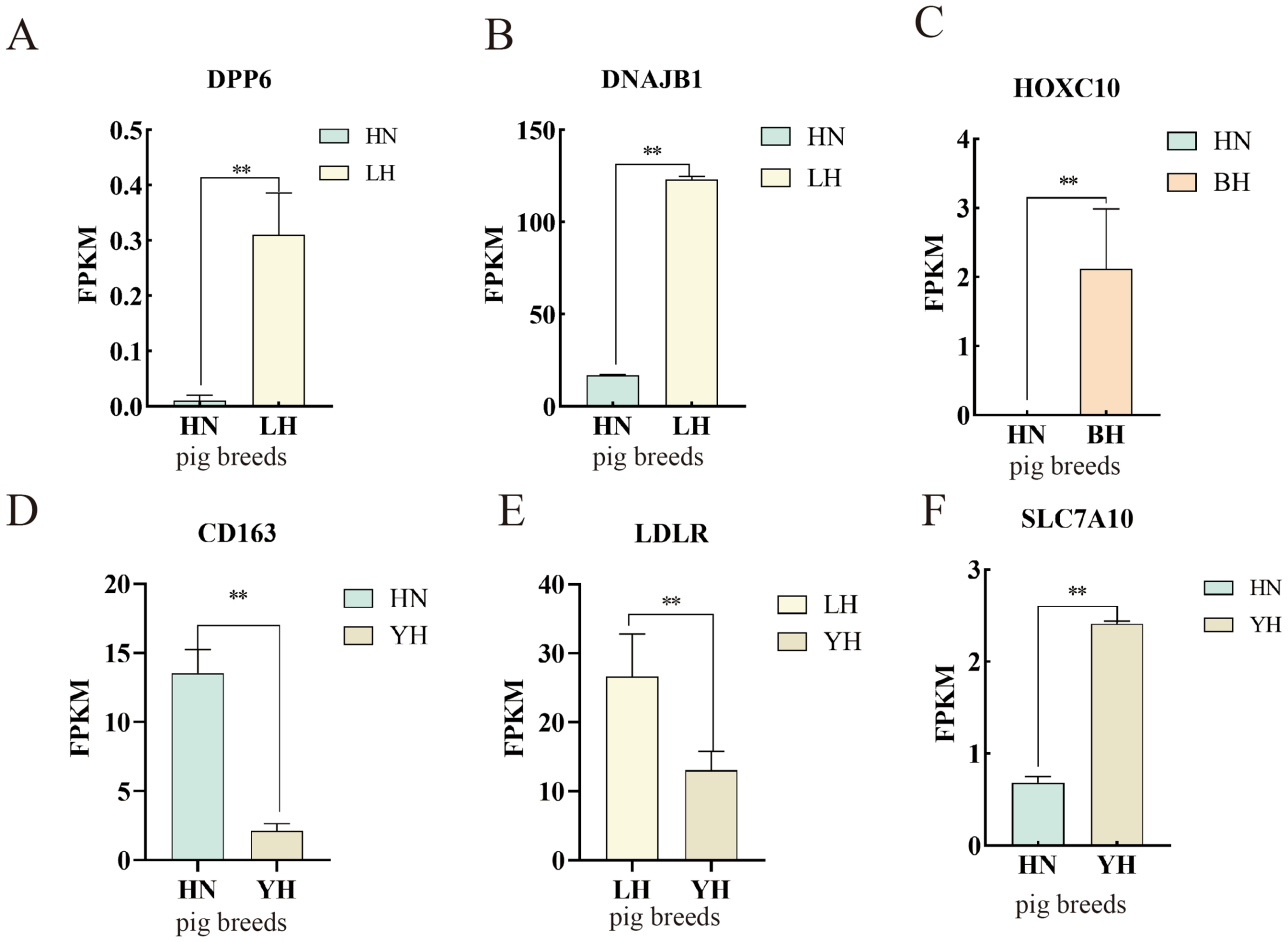
Validation of the DEGs using RNA-seq. **(A–F)** FPKM values of differentially expressed genes across different groups. ^*^p < 0.05, ^**^p < 0.01.

To verify the precision of the RNA-seq-derived gene expression profiles, we employed the qRT-PCR technique. From the pool of DEGs, we selected eight genes—*DPP6, DNAJB1, HOXC10, SLC7A10, CD163, LDLR, CPT1A,* and *WNT9B*—for validation of their relative expression levels against the RNA-seq results (Figure 10). The findings indicated that, in comparison to the HN group, the expression of *DPP6*, *DNAJB1* significantly upregulated in the LH, *SLC7A10* and *HOXC10* showed marked upregulated in the YH, with *HOXC10* was also being notably elevated in the BH (*p* < 0.01). Conversely, the YH group displayed significantly downregulated expression of*, LDLR*, *CPT1A*, and *WNT9B* compared to the LH group (*p* < 0.05). In addition, compared to HN, the expression level of *CD163* in YH is extremely downregulated (*p* > 0.05). These observations align with the previous sequencing data, reinforcing the differential expression patterns of these genes (Figure 11).

## 4 Discussion

As a crucial source of protein and essential nutrients for global consumers, the demand for pork has steadily increased, leading to heightened focus on its quality and production efficiency (11). HN pigs are a Chinese local breed known for their high litter size, tolerance to coarse feed, strong adaptability, and excellent meat quality. However, they have drawbacks such as slow growth rate and low lean meat percentage (12). In contrast, Landrace and Yorkshire pigs belong to high-quality lean meat pig breeds, characterized by rapid growth and development as well as a high lean meat percentage, which can effectively compensate for the shortcomings of HN pigs (13). In this experiment, HN pigs were used as dams to form three hybrid combinations with Yorkshire pigs, Landrace pigs, and Berkshire pigs, respectively. The backfat thickness measured between the 6th and 7th ribs, marbling score after 24 h, and leaf lard weight of HN pigs and the three crosses (BH, LH, YH) were compared. The results showed that compared to HN pigs, the backfat thickness between the 6th and 7th ribs of the three crosses (BH, LH, YH) was significantly reduced (*p* < 0.01), and the leaf lard weight was also significantly decreased (*p* < 0.01).

To further investigate the key genes that influencing SAT thickness in HN pigs and three crosses (BH, LH, YH), this study collected SAT samples from HN pigs and the three hybrid combinations for RNA-seq. The results revealed significant enrichment of key regulatory genes in SAT, including *LIPG*, *PLIN2*, *CPT1A*, *KLF9*, *CCND1*, *LDLR*, *ACSL1*, *ACLY*, and *ANGPTL4*. Furthermore, our research findings indicate that, when compared to HN, the primary differential genes in LH, BH, and YH are predominantly enriched in several key signaling pathways. These pathways include the cAMP signaling pathway, PPAR signaling pathway, cytokine-cytokine receptor interaction, PI3K-Akt signaling pathway, MAPK signaling pathway, and Hippo signaling pathway. Fat deposition is a complex process that involves fat synthesis and the formation, growth, and expansion of lipid droplets. This process is precisely regulated by multiple genes and transcription factors. According to reports, the activation of the PI3K/AKT pathway promotes fat generation, whereas its inhibition suppresses it (14). Additionally, PPARs, serving as natural ligands for fatty acids and their metabolites, regulate adipogenesis by controlling processes such as mitochondrial fatty acid oxidation, the tricarboxylic acid cycle, and oxidative phosphorylation. These processes, in turn, affect fat deposition, thereby regulating fat production and accumulation (15, 16). Furthermore, the MAPK pathway serves as a critical signaling nexus mediating extracellular signals to intracellular responses. This pathway involves core effector molecules including ERK1/2, JNK1/2/3, and p38 isoforms, which collectively orchestrate adipocyte differentiation and metabolic regulation (17). Our research results show that, compared to HN, *ANGPTL4, CPT1A, CD36, PLIN2*, and *KLF9* are significantly enriched in the PPAR signaling pathway, whereas *CSF1R, TGFB3, DUSP4, FGF18*, and *JUN* are significantly enriched in the MAPK signaling pathway. Notably, *CCND1* and *CCND2* are significantly enriched in the PI3K-Akt signaling pathway. Based on these cross-species comparative findings, we propose that hybridization of HN pigs with Berkshire, Landrace, and Yorkshire breeds induces coordinated regulation of subcutaneous adipose deposition through these differential gene expression patterns across multiple signaling pathways.

*ANGPTL4* and *PLIN2* play functionally distinct roles in regulating lipid metabolism. *ANGPTL4* functions as a multifaceted secretory protein that is synthesized across various tissue types. Conversely, *PLIN2* acts as an integral protein associated with lipid droplets and is widely recognized as a biomarker indicative of lipid droplet dynamics (18). Existing evidence has revealed that *ANGPTL4* inhibits lipoprotein lipase (LPL) through three synergistic mechanisms: it facilitates the unfolding of the enzyme, enhances proteolytic cleavage, and accelerates degradation processes. Collectively, these actions diminish the availability of catalytically active LPL pools, which are crucial for triglyceride hydrolysis (19). Additionally, the expression of *ANGPTL4* is regulated by *PPARγ* activation and positively correlates with backfat thickness in multiple pig breeds (20). Meanwhile, although lipids in lipid droplets can be catabolized through autophagy, overexpression of *PLIN2* attenuates autophagy, leading to the accumulation of lipid droplets (21). Notably, mechanistic studies have revealed coordinated regulatory interactions between *ANGPTL4* and *PLIN2*. Their combined effects promote preadipocyte differentiation and adipocyte hypertrophy, which ultimately influence the architecture and functional plasticity of adipose tissue. Experimental data from C2C12 myoblast models demonstrate that knocking down *PLIN2* significantly reduces lipid droplets and intracellular triglycerides while enhancing *β*-oxidation capacity (22). The importance of *CPT1A*, the mitochondrial gatekeeper enzyme for long-chain fatty acid transport, lies in its crucial role in regulating fatty acid oxidation during energy homeostasis (23). The metabolic significance of *CPT1A*, the mitochondrial gatekeeper enzyme for long-chain fatty acid transport, lies in its catalytic dominance in regulating fatty acid oxidation during energy homeostasis (24). Importantly, phosphorylation-modified *PLIN2* forms direct protein–protein interactions with *CPT1A*, fostering structural coupling between lipid droplets and mitochondrial networks. This spatial proximity facilitates the recruitment of adipose triglyceride lipase, thereby creating a metabolically advantageous microenvironment for efficient lipid substrate utilization (25). *KLF9* exhibits inverse correlations with depot-specific adiposity in human white adipose tissue depots (26). during the mid-phase of adipogenesis, *KLF9* occupies promoter regions of *PPARγ*, enhancing its transcriptional activation to drive terminal adipocyte maturation (27). Our study found that the expressions of *PLIN2*, *CPT1A*, KLF9 and *ANGPTL4* in the BH, LH, and YH groups all significantly significant downregulation compared to HN. Furthermore, when compared the LH group, the expressions of *CPT1A* in the BH and YH groups also demonstrated a downward trend. Based on these results, we suggest that crossing HN pigs with Berkshire, Landrace, and Yorkshire pigs leads to reduced fat deposition abilities in the BH, LH, and YH groups. However, the LH group seems to have a relatively stronger fat deposition ability than the other two groups.

*CCND1* and *CCND2* positively regulate adipogenesis by modulating the cell cycle and fatty acid synthesis. They play crucial roles in the early differentiation of adipocytes interact with metabolic transcription factors, particularly *PPARγ*, along with *CCNG1* and *CCNG2*, to collectively regulate adipocyte differentiation (14, 28). In this study, the expression levels of *CCND1* and *CCND2* in BH, LH, and YH were significantly upregulated compared to HN. This finding suggests that *CCND2* and *CCND2* play a regulatory role in subcutaneous fat deposition in YH. Additionally, LIPG is crucial for lipoprotein metabolism, cytokine expression, and cellular lipid composition, with a genetic reduction in its function linked to elevated triglyceride levels in humans. ACSLs facilitate the acylation of fatty acids into long-chain acyl-CoA (LCA-CoA), which is utilized for *β*-oxidation or esterification, thus promoting adipogenic differentiation and fat accumulation. Notably, during the differentiation of porcine subcutaneous preadipocytes, there is an increase in the expression of *ACSL1*. In this study, the expression levels of *CCND1* and *CCND2* in BH, LH, and YH were significantly upregulated compared to HN. This finding suggests that *CCND2* and *CCND2* regulate subcutaneous fat deposition in YH. Additionally, *LIPG* is crucial for lipoprotein metabolism, cytokine expression, and cellular lipid composition, with a genetic reduction in its function is associated with elevated triglyceride levels in humans (29). *ACSLs* facilitate the catalyze of fatty acids into long-chain acyl-CoA (LCA-CoA), which is utilized for β-oxidation or esterification, thereby promoting adipogenic differentiation and fat accumulation Notably, during the differentiation of porcine subcutaneous preadipocytes, the expression of *ACSL1* increases (30). *CD36* maintains fatty acid homeostasis during adipogenesis, being induced by p38 and AMPK kinases and transcriptionally regulated by *PPARγ* (14). *CD36* enhances fatty acid transport by binding to them and may work synergistically with FATP (31). *ACSL1* in mitochondria plays a key role in fatty acid oxidation and regulates AMPK activation in adipocytes (32). Our research results indicate that the expression of *ACLY, LDLR, ACSL1*, and *LIPG* is upregulated in the LH group. Based on this, we hypothesize that the LH group regulates fat deposition by modulating fatty acid synthesis, transport, and breakdown.

Previous studies have reported that *PGAM2* expression significantly influences growth rate, feed conversion, and post-slaughter meat traits, with higher expression levels negatively correlating with beef tenderness (33, 34). The results of this study indicate that, compared to HN, the DEGs in BH were mainly enriched in pathways related to alpha-linolenic acid metabolism, carbon metabolism, glycine, serine, and threonine metabolism, as well as arachidonic acid metabolism. Notably, the expressions of *AGXT, ALAS2*, *PGAM2*, *PSPH*, and *CBS*, which are related to glycine, serine, and threonine metabolism, were also upregulated. In this study, compared to HN, the expressions of *ACAA2, ACCA, ACADL, FASN, SCD, ELOVL5*, and *ELOVL6* in fatty acid metabolism-related pathways were all upregulated in LH. Previous research results have shown a positive correlation between the expressions of *FASN, SCD, ACACA*, and *ELOVL6* genes and backfat thickness (35). In addition, the *SCD* gene directly catalyzes the conversion of stearic acid to oleic acid and synergizes with peroxisome proliferator-activated receptor and other signaling pathway genes, potentially promoting subcutaneous fat deposition by regulating lipid metabolism (36). Studies of the *SCD* gene promoter polymorphism (rs80912566) in Duroc pigs have shown that the T allele is closely associated with high *SCD* expression and increased levels of monounsaturated fatty acids (37). Therefore, we speculate that the upregulation of genes such as *FASN, SCD,* and *ELOVL6* in the LH group may lead to increased backfat thickness, which in turn promotes subcutaneous fat deposition. When YH is compared with HN, genes related to fatty acid elongation and carbon metabolism generally show an upregulation trend. The expressions of fatty acid elongases (*ELOVL4, ELOVL5*, and *ELOVL6*) were upregulated. Among them, *ELOVL6* catalyzes fatty acid elongation and is involved in regulating fat deposition (38). Therefore, in this study, the *ELOVL6* gene positively regulated subcutaneous fat deposition in YH.

It is noteworthy that, although some differentially expressed genes are upregulated in the three hybrid combinations compared to HN, the upregulation of genes involved in fatty acid metabolism does not necessarily lead to higher fat deposition. Fat deposition is a complex biological process regulated by multiple factors (39). Although fatty acid metabolism plays an important role in this process, its impact is not singular and direct. Furthermore, the regulation of fatty acid metabolism may involve multiple biological processes, including fatty acid synthesis, catabolism, transport, and storage. The balance among these processes determines the ultimate amount of fat deposition (40). Therefore, we speculate that even if certain genes involved in fatty acid metabolism are upregulated in hybrid offspring, they may contribute to reducing fat accumulation by promoting processes such as fatty acid catabolism or transport.

## 5 Conclusion

Using RNA-seq analysis, we investigated the differences in fat deposition among the offspring of HN pigs crossed with different lean breeds. The study revealed that differentially expressed genes (*LIPG, PLIN2, CPT1A, KLF9, CCND1, CCND2, LDLR, ACSL1, ACLY,* and *ANGPTL4*) may regulate subcutaneous fat deposition in the HN, BH, LH, and YH groups through the PI3K-Akt, PPAR, and MAPK signaling pathways. These findings enhance our understanding of the genetics of adipose tissue in crossbred pigs, potentially benefiting pork quality and health.

## Data Availability

The data presented in the study are deposited in the figshare database repository, and the access link is https://doi.org/10.6084/m9.figshare.28077401.

## Footnotes

^1^ https://cutadapt.readthedocs.io/en/stable/

^2^ https://daehwankimlab.github.io/hisat2/

^3^ http://ccb.jhu.edu/software/stringtie/

^4^ http://ccb.jhu.edu/software/stringtie/gffcompare.shtml

^5^ http://geneontology.org/

^6^ http://www.genome.jp/kegg/

^7^ https://string-db.org

## References

[1] Wang W Wang D Zhang X Liu X Niu X Li S. Comparative transcriptome analysis of longissimus dorsi muscle reveal potential genes affecting meat trait in Chinese indigenous Xiang pig. Sci Rep. (2024) 14:8486. doi: 10.1038/s41598-024-58971-2, PMID: 38605105 PMC11009340

[2] Han Y Tan T Li Z Ma Z Lan G Liang J. Identification of selection signatures and loci associated with important economic traits in Yunan black and Huainan pigs. Genes. (2023) 14:655. doi: 10.3390/genes14030655, PMID: 36980926 PMC10048629

[3] Zhang D Wu W Huang X Xu K Zheng C Zhang J. Comparative analysis of gene expression profiles in differentiated subcutaneous adipocytes between Jiaxing black and large white pigs. BMC Genomics. (2021) 22:61. doi: 10.1186/s12864-020-07361-9, PMID: 33468065 PMC7814706

[4] Chen Q Zhang W Cai J Ni Y Xiao L Zhang J. Transcriptome analysis in comparing carcass and meat quality traits of Jiaxing black pig and Duroc × Duroc × Berkshire × Jiaxing black pig crosses. Gene. (2022) 808:145978. doi: 10.1016/j.gene.2021.145978, PMID: 34592352

[5] Hang J Wang J Ma C Wang W Wang H Jiang Y. Comparative transcriptomic analysis of mRNAs, miRNAs and lncRNAs in the longissimus dorsi muscles between fat-type and lean-type pigs. Biomol Ther. (2022) 12:1294. doi: 10.3390/biom12091294, PMID: 36139132 PMC9496231

[6] Liu Y Yang Y Wu R Gao CC Liao X Han X. mRNA m5C inhibits adipogenesis and promotes myogenesis by respectively facilitating YBX2 and SMO mRNA export in ALYREF-m5C manner. Cell Mol Life Sci. (2022) 79:481. doi: 10.1007/s00018-022-04474-0, PMID: 35962235 PMC11072269

[7] Deng X Zhang Y Song G Fu Y Chen Y Gao H. Integrative analysis of transcriptomic and lipidomic profiles reveals a differential subcutaneous adipose tissue mechanism among Ningxiang pig and Berkshires, and their offspring. Animals. (2023) 13:3321. doi: 10.3390/ani13213321, PMID: 37958077 PMC10647668

[8] Li Y Jia M Chen J Liu F Ren Q Yan X. A comparative metabolomics study of the potential marker compounds in feces from different hybrid offspring of Huainan pigs. Animals. (2024) 14:3282. doi: 10.3390/ani14223282 39595336 PMC11591501

[9] Gene Ontology Consortium. The gene ontology resource: enriching a gold mine. Nucleic Acids Res. (2021) 49:D325–34. doi: 10.1093/nar/gkaa1113, PMID: 33290552 PMC7779012

[10] Kanehisa M Furumichi M Sato Y Ishiguro-Watanabe M Tanabe M. KEGG: integrating viruses and cellular organisms. Nucleic Acids Res. (2021) 49:D545–51. doi: 10.1093/nar/gkaa970, PMID: 33125081 PMC7779016

[11] Zhao X Jia W Wang J Wang S Zheng Q Shan T. Identification of a candidate gene regulating intramuscular fat content in pigs through the integrative analysis of transcriptomics and proteomics data. J Agric Food Chem. (2023) 71:19154–64. doi: 10.1021/acs.jafc.3c05806, PMID: 37987700

[12] Newcom DW Baas TJ Schwab CR Stalder KJ. Genetic and phenotypic relationships between individual subcutaneous backfat layers and percentage of longissimus intramuscular fat in Duroc swine. J Anim Sci. (2005) 83:316–23. doi: 10.2527/2005.832316x, PMID: 15644502

[13] Zhang P Li Q Wu Y Zhang Y Zhang B Zhang H. Identification of candidate genes that specifically regulate subcutaneous and intramuscular fat deposition using transcriptomic and proteomic profiles in Dingyuan pigs. Sci Rep. (2022) 12:2844. doi: 10.1038/s41598-022-06868-3, PMID: 35181733 PMC8857214

[14] Song Y Zhang J Jiang C Song X Wu H Zhang J. FOXO1 regulates the formation of bovine fat by targeting CD36 and STEAP4. Int J Biol Macromol. (2023) 248:126025. doi: 10.1016/j.ijbiomac.2023.126025, PMID: 37506793

[15] Xu D Zhuang S Chen H Jiang M Jiang P Wang Q. IL-33 regulates adipogenesis via Wnt/β-catenin/PPAR-γ signaling pathway in preadipocytes. J Transl Med. (2024) 22:363. doi: 10.1186/s12967-024-05180-0, PMID: 38632591 PMC11022325

[16] Hong L Sun Z Xu D Li W Cao N Fu X. Transcriptome and lipidome integration unveils mechanisms of fatty liver formation in Shitou geese. Poult Sci. (2024) 103:103280. doi: 10.1016/j.psj.2023.103280, PMID: 38042038 PMC10711516

[17] Zhang C Wang L Qin L Luo Y Wen Z Vignon AS. Overexpression of GPX2 gene regulates the development of porcine preadipocytes and skeletal muscle cells through MAPK signaling pathway. PLoS One. (2024) 19:e0298827. doi: 10.1371/journal.pone.0298827, PMID: 38722949 PMC11081289

[18] Dahlhoff M Camera E Picardo M Zouboulis CC Chan L Chang BH. PLIN2, the major perilipin regulated during sebocyte differentiation, controls sebaceous lipid accumulation in vitro and sebaceous gland size in vivo. Biochim Biophys Acta. (2013) 1830:4642–9. doi: 10.1016/j.bbagen.2013.05.016, PMID: 23688400 PMC3998206

[19] Landfors F Chorell E Kersten S. Genetic mimicry analysis reveals the specific lipases targeted by the ANGPTL3-ANGPTL8 complex and ANGPTL4. J Lipid Res. (2023) 64:100313. doi: 10.1016/j.jlr.2022.100313, PMID: 36372100 PMC9852701

[20] Blücher C Iberl S Schwagarus N Müller S Liebisch G Höring M. Secreted factors from adipose tissue reprogram tumor lipid metabolism and induce motility by modulating PPARα/ANGPTL4 and FAK. Mol Cancer Res. (2020) 18:1849–62. doi: 10.1158/1541-7786.MCR-19-1223, PMID: 32859692

[21] Zhao Y Chen S Yuan J Shi Y Wang Y Xi Y. Comprehensive analysis of the lncRNA-miRNA-mRNA regulatory network for intramuscular fat in pigs. Genes. (2023) 14:168. doi: 10.3390/genes14010168, PMID: 36672909 PMC9859044

[22] Zhu R Chen S. Proteomic analysis reveals semaglutide impacts lipogenic protein expression in epididymal adipose tissue of obese mice. Front Endocrinol. (2023) 14:1095432. doi: 10.3389/fendo.2023.1095432 PMC10070826 37025414

[23] Bosma M Hesselink MK Sparks LM Timmers S Ferraz MJ Mattijssen F. Perilipin 2 improves insulin sensitivity in skeletal muscle despite elevated intramuscular lipid levels. Diabetes. (2012) 61:2679–90. doi: 10.2337/db11-1402, PMID: 22807032 PMC3478528

[24] Liu L Wang YD Wu J Cui J Chen T. Carnitine palmitoyl transferase 1A (CPT1A): a transcriptional target of PAX3-FKHR and mediates PAX3-FKHR-dependent motility in alveolar rhabdomyosarcoma cells. BMC Cancer. (2012) 12:154. doi: 10.1186/1471-2407-12-154, PMID: 22533991 PMC3453510

[25] Meng Y Guo D Lin L Zhao H Xu W Luo S. Glycolytic enzyme PFKL governs lipolysis by promoting lipid droplet-mitochondria tethering to enhance β-oxidation and tumor cell proliferation. Nat Metab. (2024) 6:1092–107. doi: 10.1038/s42255-024-01047-2, PMID: 38773347

[26] Lee JE Schmidt H Lai B Ge K. Transcriptional and epigenomic regulation of adipogenesis. Mol Cell Biol. (2019) 39:e00601–18. doi: 10.1128/MCB.00601-18, PMID: 30936246 PMC6517598

[27] Pei H Yao Y Yang Y Liao K Wu JR. Krüppel-like factor KLF9 regulates PPARγ transactivation at the middle stage of adipogenesis. Cell Death Differ. (2011) 18:315–27. doi: 10.1038/cdd.2010.100, PMID: 20725087 PMC3131894

[28] Sun M Gao J Meng T Liu S Chen H Liu Q. Cyclin G2 upregulation impairs migration, invasion, and network formation through RNF123/Dvl2/JNK signaling in the trophoblast cell line HTR8/SVneo, a possible role in preeclampsia. FASEB J. (2021) 35:e21169. doi: 10.1096/fj.202001559RR, PMID: 33205477

[29] Xie P Guo M Xie JB Xiao MY Qi YS Duan Y. Effects of heat-processed gynostemma pentaphyllum on high-fat diet-fed mice of obesity and functional analysis on network pharmacology and molecular docking strategy. J Ethnopharmacol. (2022) 294:115335. doi: 10.1016/j.jep.2022.115335, PMID: 35513215

[30] Jiang Y Wang W Wang H Zhang X Kong Y Chen YQ. ACSL1 positively regulates adipogenic differentiation. Biochem Biophys Res Commun. (2024) 735:150865. doi: 10.1016/j.bbrc.2024.150865 39442449

[31] McTavish PV Mutch DM. Omega-3 fatty acid regulation of lipoprotein lipase and FAT/CD36 and its impact on white adipose tissue lipid uptake. Lipids Health Dis. (2024) 23:386. doi: 10.1186/s12944-024-02376-7, PMID: 39567971 PMC11580630

[32] Yang M Ge J Liu YL Wang HY Wang ZH Li DP. Sortilin-mediated translocation of mitochondrial ACSL1 impairs adipocyte thermogenesis and energy expenditure in male mice. Nat Commun. (2024) 15:7746. doi: 10.1038/s41467-024-52218-4, PMID: 39232011 PMC11374900

[33] Fontanesi L Davoli R Nanni Costa L Beretti F Scotti E Tazzoli M. Investigation of candidate genes for glycolytic potential of porcine skeletal muscle: association with meat quality and production traits in Italian large white pigs. Meat Sci. (2008) 80:780–7. doi: 10.1016/j.meatsci.2008.03.022, PMID: 22063597

[34] Antonelo DS Gómez JFM Silva SL Beline M Zhang X Wang Y. Proteome basis for the biological variations in color and tenderness of longissimus thoracis muscle from beef cattle differing in growth rate and feeding regime. Food Res Int. (2022) 153:110947. doi: 10.1016/j.foodres.2022.110947, PMID: 35227471

[35] Du L Li K Chang T An B Liang M Deng T. Integrating genomics and transcriptomics to identify candidate genes for subcutaneous fat deposition in beef cattle. Genomics. (2022) 114:110406. doi: 10.1016/j.ygeno.2022.110406 35709924

[36] Valdés-Hernández J Ramayo-Caldas Y Passols M Criado-Mesas L Castelló A Sánchez A. Identification of differentially expressed genes and polymorphisms related to intramuscular oleic-to-stearic fatty acid ratio in pigs. Anim Genet. (2025) 56:e13491. doi: 10.1111/age.13491, PMID: 39593270

[37] Estany J Ros-Freixedes R Tor M Pena RN. A functional variant in the stearoyl-CoA desaturase gene promoter enhances fatty acid desaturation in pork. PLoS One. (2014) 9:e86177. doi: 10.1371/journal.pone.0086177, PMID: 24465944 PMC3896438

[38] Junjvlieke Z Khan R Mei C Cheng G Wang S Raza SHA. Effect of ELOVL6 on the lipid metabolism of bovine adipocytes. Genomics. (2020) 112:2282–90. doi: 10.1016/j.ygeno.2019.12.024 31901374

[39] Morigny P Boucher J Arner P Langin D. Lipid and glucose metabolism in white adipocytes: pathways, dysfunction and therapeutics. Nat Rev Endocrinol. (2021) 17:276–95. doi: 10.1038/s41574-021-00471-8, PMID: 33627836

[40] Jeon YG Kim YY Lee G Kim JB. Physiological and pathological roles of lipogenesis. Nat Metab. (2023) 5:735–59. doi: 10.1038/s42255-023-00786-y, PMID: 37142787

